# The Effect of a Dilemma on the Relationship Between Ability to Identify the Criterion (ATIC) and Scores on a Validated Situational Interview

**DOI:** 10.3389/fpsyg.2021.674815

**Published:** 2021-07-27

**Authors:** Gary P. Latham, Guy Itzchakov

**Affiliations:** ^1^Rotman School of Management, University of Toronto, Toronto, ON, Canada; ^2^Department of Human Services, University of Haifa, Haifa, Israel

**Keywords:** situational interview, employee selection, recruitment, human resource management, assessment

## Abstract

Four experiments were conducted to determine whether participants' awareness of the performance criterion on which they were being evaluated results in higher scores on a criterion valid situational interview (SI) where each question either contains or does not contain a dilemma. In the first experiment there was no significant difference between those who were or were not informed of the performance criterion that the SI questions predicted. Experiment 2 replicated this finding. In each instance the SI questions in these two experiments contained a dilemma. In a third experiment, participants were randomly assigned to a 2 (knowledge/no knowledge provided of the criterion) X 2 (SI dilemma/no dilemma) design. Knowledge of the criterion increased interview scores only when the questions did *not* contain a dilemma. The fourth experiment revealed that including a dilemma in a SI question attenuates the ATIC-SI relationship when participants must identify rather than be informed of the performance criterion that the SI has been developed to assess.

## Introduction

The employment interview has long been known to be a deeply flawed method for selecting individuals (Wagner, 1949; Ulrich and Trumbo, 1965). In many instances, it is tantamount to little more than an unstructured conversation between two or more individuals. The result is a selection technique that has low reliability and validity.

In the 1980s, two structured interview techniques were developed that overcame these issues. All job applicants are asked the same job-related questions derived from a systematic job analysis. The result is two reliable, valid methods for interviewing candidates. These two methods are the situational interview (SI; Latham, 1989) and the patterned behavior description interview (PBDI; Janz, 1989). The premise underlying the SI is that intentions predict behavior. Applicants are asked to respond to questions derived from a job analysis by explaining what they would do in sundry situations. The premise underlying the PBDI is that among the best predictors of future behavior is an individual's past behavior. A meta-analysis of the research on the effectiveness of these two interview techniques revealed that the SI has higher overall mean criterion-related validity (*M* = 0.23) compared to the PBDI (*M* = 0.18) for predicting an individual's job performance (Culbertson et al., 2017). Similarly, Levashina et al. (2014), in a review of the literature, found that past behavior interview questions had lower group differences than situational interviews (*d* = 0.10, *d* = 0.20, respectively). Hence, the present research focused on the SI and the extent to which knowing, that is, being informed of vs. identifying, the job performance criterion or criteria the SI was developed to assess improves an individual's performance in this interview.

### Ability to Identify Criteria (ATIC)

Kleinmann (1993) and colleagues (e.g., Ingold et al., 2015) found that the ability to correctly identify the job criterion that is being predicted in a criterion valid SI increases both an individual's score on the SI and subsequent performance on the job. They also made this claim for assessment centers (e.g., Jansen et al., 2011). In short, they concluded it is ability to identify criteria (ATIC) that not only increases an individual's performance in these two selection procedures, but predicts performance on the job as well. This is because ATIC is said to enable job candidates to “provide evaluation relevant answers in the interview, as well as demonstrate evaluation relevant behaviors on the job” (Ingold et al., 2015, p. 389). However, with regard to assessment centers, it is noteworthy that ratings of performance on non-transparent dimensions were shown to be more criterion valid than ratings from assessment centers with transparent dimensions (Ingold et al., 2016). Based on their research, König et al. (2007) similarly concluded that selection interviews should not be made transparent.

Findings from research on ATIC have far reaching implications for human resource management. A downside is that the research suggests that, similar to self-report personality tests, the SI is susceptible to applicants “faking” their responses. Faking may be especially problematic regarding ATIC if applicants take the time to discover an organization's values/culture, strategy, and desired job competencies prior to applying for a job, as this would increase their likelihood of being able to identify the criterion or criteria on which they will be assessed.

The upsides of an individual's ability to identify the job performance criterion being assessed arguably out-weigh this downside. ATIC is an individual difference variable. Hence, ATIC is advantageous for some job applicants because, as noted earlier, those who score high on this measure “are more likely to discern criteria for success both in the SI and on the job” (Ingold et al., 2015, p. 389). This, in turn, not only enables applicants to perform well in a SI, but it also enables them to “demonstrate evaluation-relevant behaviors on the job” (p. 389). Ingold et al. gave the example of an individual who recognizes the importance of cooperativeness as a performance criterion and then emphasizes cooperation when responding to a SI question and subsequently making “efforts to cooperate (rather than compete) with coworkers on the job (p. 389).

ATIC has been defined as a form of context-specific social effectiveness. Tangential evidence supporting the Ingold et al. (2015) finding that ATIC affects the relationship between the SI and job performance can be found in the study by Sue-Chan and Latham (2004). They found that emotional intelligence mediated the relationship between the SI and team work skills. This finding is consistent with Griffin's (2014) assertion that social understanding, as noted above, predicts ATIC scores.

Melchers et al. (2012) stated that there are two possible reasons why ability to identify the performance criterion that is being assessed in an interview results in better performance. The first possibility is that some candidates have the ability to provide more accurate ideas than others. A second possibility is that some candidates merely generate more ideas in general regarding the performance dimensions that are being assessed. Melchers et al.'s (2012) analysis revealed that it is the first possibility, namely, the ability to provide more *accurate* ideas of what is being assessed that predicts better performance in the job interview.

It might be argued, based on Kleinmann et al's. studies (e.g., Kleinmann et al., 2011), that ATIC is a proxy for general mental ability (GMA). Given GMA is among the strongest single predictor of job performance, it might affect an individual's ability to infer what is being assessed by a SI. However, in a criterion-related validity study involving managers, where the criterion was an assessment of teamwork skills, the correlation between the SI and GMA was not significant (Sue-Chan and Latham, 2004). This finding is consistent with a series of meta-analyses conducted by Cortina et al. (2000). They found that highly structured interviews have incremental validity beyond cognitive ability. Furthermore, a meta-analysis revealed a weak relationship (*r* = 0.09) between the SI and cognitive ability (Culbertson et al., 2017).

There are reasons to question the findings on ATIC as an explanation for the criterion related validity of an SI. In the domain of training, supervisors who were given the learning points that they were asked to demonstrate performed no better than those in the control group where this information was not provided (Latham and Saari, 1979). In short, knowledge alone was not sufficient for bringing about a desired change in behavior.

In response to Kleinmann's (1993) and Griffin's (2014) call for research on ATIC under both transparent and non-transparent performance criterion conditions, the purpose of the present research was to examine the possibility that the alleged benefit of the ATIC for answering SI questions is based on inappropriate research methodology, namely, the failure to include a dilemma in each SI question. To do so, we first briefly discuss the correct development of a SI. We then discuss the methodology used by Ingold et al. (2015) and Oostrom et al. (2016) to develop an SI. Finally, we present the results of our four experiments. In the first two, individuals were informed of the performance criterion that the SI predicted. In the third experiment, interview scores on SI questions that did vs. did not contain a dilemma were examined. In the fourth experiment, a direct measure of ATIC was employed. We did so to expand previous work by testing if ATIC increases scores on SI questions that include a dilemma relative to SI questions that do not include a dilemma, as was found in previous work (e.g., Ingold et al., 2015; Oostrom et al., 2016). The direct measure of ATIC was consistent with extant ATIC procedures (e.g., Oostrom et al., 2016).

### The Situational Interview

Consistent with Campion, Palmer and Campion (1997) typology, the SI is a structured interview in that the questions are based on a job analysis, the same questions are asked of each interviewee, prompting an individual is not allowed, notes are taken by two or more interviewers, and the same interviewers are used across interviewees. The interviewers use a predetermined scoring guide to evaluate each interviewee's answer to an interview question.

The premise of the SI is that intentions predict behavior (Latham et al., 1980; Latham, 1989). Intentions are “a representation of a future course of action to be performed…a proactive commitment to bringing them (future actions) about” (Bandura, 2000, p. 5). Intentions are generally viewed as the direct motivational instigator of behavior (Klehe and Latham, 2006; Locke and Latham, 2013).

The SI (Latham, 1989; Latham and Sue-Chan, 1999) has five distinct features. First, as noted earlier, it is based on a systematic job analysis, typically the critical incident technique (Flanagan, 1954). Consistent with Wernimont and Campbell's (1968) argument to develop predictors consistent with the performance criteria, the performance criteria (e.g., Behavioral Observation Scales/BOS; Latham and Wexley, 1977) and the SI questions are developed from the same job analysis.

Second, the context, behavior, and outcomes described in a critical incident are turned into a question: “What would you do in this situation?” Each SI question contains a dilemma.

In a valid SI interview, the dilemma confronting an individual is having to choose between two or more exclusive courses of action (Latham and Sue-Chan, 1999; Levashina et al., 2014). The purpose of the dilemma is to “force” applicants to state their actual intentions rather than offer socially desirable responses (Latham, 1989; Sue-Chan and Latham, 2004).

An example of a question that only assesses a future intention is: “As you are crossing a busy street, your aging parent, who is nearing the middle of the road, calls out to you for assistance. What would you do in this situation?” Note that the question does not contain a dilemma.

In contrast, an example of a SI question that contains a dilemma is as follows: “As you are crossing a busy street your aging parent, who is nearing the middle of the road, calls out to you for assistance. As you turn in her direction, a gust of wind blows the lottery ticket worth a million dollars out of your hand down the street. What would you do in this situation?”

The presentation of a dilemma to interviewees, in this instance helping your mother vs. going after the lottery ticket, is critical to the development of a SI because, as noted earlier, the underlying premise is that an individual's intentions predict behavior. If an SI question does not contain a dilemma, the answer to an interview question may be a response to what the interviewee infers the interviewer hopes to hear. Hence the dilemma is a core aspect of the SI (Levashina et al., 2014)^1^. In short, the importance of a dilemma to differentiate an SI from a non SI question cannot be over-emphasized (Latham, 1989; Latham and Sue-Chan, 1999; Klehe and Latham, 2005). Nevertheless, in conducting their meta-analysis, Taylor and Small (2002) reported a great deal of unexplained variance across SI studies due to the fact that many studies claiming to be an SI did not include a dilemma:

“We noticed rather heterogeneous approaches to how questions and answer rating scales were developed among primary studies. Examples of situational questions developed by Latham et al. suggest that those authors not only pose questions as hypothetical dilemmas, but that these dilemmas typically involve choices between two competing values. In contrast, other researchers have developed situational questions which neither present dilemmas nor focus on values” (Taylor and Small, 2002, p. 290).

Likewise, in conducting their meta-analysis, Huffcutt et al. (2004, p. 269) found that “a majority of the situational studies in the current interview literature include questions that do not have a dilemma.” Examples of studies that failed to include a dilemma include Campion et al. (1994) and Pulakos and Schmitt (1995)^2^. Low validity coefficients were obtained in both studies. In addition, Levashina et al. (2014), in their review of the literature, concluded that although a dilemma is a core aspect of valid situational interview questions (Latham et al., 1980), many researchers have used situational interview questions that did not contain dilemmas.

Third, a behavioral scoring guide is developed by subject matter experts (e.g., supervisors, customers) to aid the interviewers in scoring the response to each question. This is done to minimize interviewer biases in the scoring of responses, and to increase interrater reliability.

Fourth, the scoring of each individual's response to an SI question is conducted by a panel of two or more individuals. Each member of the panel scores each answer independently.

Fifth, a pilot study is conducted to determine whether there is variability in the responses to each SI question. If most people give a correct/incorrect response, the question is discarded. As Guion (1998, p. 614) commented, “the explicit provision of a pilot study for the SI is noteworthy because people who would never dream of developing written tests without pilot studies do not hesitate to develop interview guides without them. Building a psychometric device without pilot studies displays unwarranted arrogance—or ignorance of the many things that can go wrong.” An example of adhering to this guideline can be found in Klehe and Latham (2005). Specifically, a pilot study (*n* = 31) was conducted to determine if there was variability in the responses to the questions before the criterion validity study was conducted and to determine whether there was interrater reliability when using the behavioral scoring guide.

### Ingold et al. (2015) Study

Ingold et al.'s study was designed to answer the following question: Why do situational interviews predict job performance? Their answer was the “interviewee's ability to identify criteria” (p. 388). In their study, no job analysis was conducted to develop the performance criteria or the SI questions to predict them. Instead, Ingold et al. focused on what they called a management trainee position as the targeted job. Their experiment involved 97 current and prospective University graduates who were employed or had been recently employed. Over half (55%) held a Master's or a comparable degree. Two interviewers, as a panel, conducted a mock interview to assess assertiveness, perseverance, and organizing behavior. Each participant's supervisor assessed the individual's performance, using a 7-point scale, on five items from Williams and Anderson (1991) and five items from Jansen et al. (2013) for assessing a general manager. Because the scores on the two scales were highly correlated, a composite score was computed. Examples of items from the two respective scales are: “Demonstrates expertise in all job-related tasks”; “adequately completes assigned duties.”

Rather than develop SI questions, Ingold et al. contacted authors of previous SI studies for permission to adapt SI questions for their study along with the respective behavioral scoring guide for each question. Several questions failed to include a dilemma (see Box 1). Consequently, only two of the three concurrent validity coefficients were significant with supervisory ratings of job performance. Specifically, perseverance, [*r* = 0.23, *p* < 0.05], organizing behaviors, [*r* = 0.30, *p* < 0.01], and assertiveness, [*r* = 0.11, *p* = 0.27]. The correlation between ATIC and SI performance was significant [*r* = 0.23, *p* < 0.05]. If Ingold et al. had followed step 5, namely, conduct a pilot study to determine whether there is variability in the responses, and in addition only presented questions that contained a dilemma, all three correlation coefficients might have been valid, and the magnitude of the validity coefficients might have been higher.

Box 1 **SI Question used by Ingold et al. (2015).**

Perseverance: Imagine you're finding the first months at your new job very difficult. The tasks you are assigned are very demanding, and you think your boss isn't entirely satisfied with your work. Please describe briefly how you would behave in this situation.There is no dilemma in this question.

Following the SI, the participants completed a questionnaire where they were told to write the criterion that they believed an SI question was assessing, and to provide a behavioral example. Ingold and a Master's student rated the accuracy of each participant's responses on a 4-point scale (i.e., no fit, limited fit, a moderate fit, fits completely). They then tested whether people with high scores on ATIC performed better on the questions corresponding to a specific dimension, whether ATIC predicted supervisory assessments of job performance, and whether it explained incremental variance in job performance beyond the SI. They obtained supporting evidence in each instance. Finally, Ingold et al. found that the SI did not predict performance on the job when ATIC was controlled. Thus, the hypothesis tested in our first experiment is that individuals who are made aware of the performance criterion obtain a significantly higher score on a criterion valid SI than those who are not informed.

## Overview of Present Experiments

The present research used a predictively valid SI that was developed by Klehe and Latham (2005) for assessing the teamwork skills of applicants to an MBA program. The uncorrected *r* is 0.41 (*p* < 0.05). The MBA program requires much of the course work to be performed in teams, making teamwork skills a critical prerequisite for a student to receive an MBA degree.

Both the teamwork criterion and the SI questions were derived from a systematic job analysis, the critical incident technique (Flanagan, 1954). Each SI question developed by Klehe and Latham (2005) contained a dilemma (see Box 2). As was the case in the Ingold et al. (2015) study, a behavioral scoring guide was developed for each SI question, and the responses to the questions were scored by a panel (Klehe and Latham, 2005). A pilot study was then conducted with MBA students who had not taken part in the criterion validation study. Questions for which agreement on the scoring could not be reached, as well as questions that revealed a lack of variance in the responses to them were discarded. This process resulted in nine SI questions. Each answer to the SI question was rated on a Likert-type scale ranging from 1 to 5. In all four of our experiments, we calculated the score for each SI question by calculating the average score of the two raters.

Box 2 **An SI question used by Klehe and Latham (2005).**

Your group is working on a very important project. All of you want to achieve a good grade. You have a tight deadline. One member of your group was especially successful in this area last term. Supported by two other group members, she takes the lead on your group project. She keeps the minutes and controls the flow of information during the discussion. However, you have the strong impression that she only records ideas supportive of her position and makes decisions on issues without consulting with others. What would you do?

The dilemma is between meeting a tight deadline and attaining a high grade versus the importance of ensuring that the input of others on the team is taken into account.

In the first three experiments, participants were informed of the performance criterion that was being assessed by the SI. This was done to test whether providing knowledge of the performance criterion enables individuals to provide relevant responses to the SI questions and hence receive higher scores than those in the control condition.

In the third experiment, participants were randomly assigned to conditions that did or did not include a dilemma, and did or did not include information about the performance criterion that was being assessed. This was done to determine whether knowledge given to participants of the performance criterion the SI questions assess increases SI scores when a dilemma is/is not contained in a question. In the fourth experiment, we conceptually replicated the results of the third experiment. Specifically, we examined whether an individual's ability to identify the performance criteria an SI was developed to predict increases interview scores only when the questions do not include a dilemma.

## Experiment 1

The hypothesis tested in this experiment is that being informed of the performance criterion that is being assessed leads to higher scores on SI questions that contain a dilemma than the scores in a control condition where this information was not provided.

### Method

#### Participants

Participants (*n* = 108, *M*_age_ = 35.09, *SD*_*age*_ = 12.09, 45.4% female), recruited through CrowdFlower, an online subject pool platform, responded to the nine SI interview questions on the online data collection tool, Qualtrics. They did so in exchange for a monetary payment. The study lasted ~15 min. Twenty-six percent of the participants were in between jobs, 53.3% were employed full-time (i.e., worked more than 35 h per week), and 20.6% held a part-time job. On average, they had 11.15 (*SD* = 10.49) years of job experience. Twenty-seven percent had an Associate Degree, 52.5% held a Bachelor's Degree, 10.9% held either an MA or a Ph.D., and 9.9% had a Professional Degree. Of those currently employed, 5.6% of the participants worked in research and education, 4.6% in banking and insurance, 5.6% in the service industry, 6.5% in sales, 7.4% in the public sector, and 13.9% were employed in other industries. No participant was excluded from the data analysis.

Power analysis using GPower (Faul et al., 2007) indicated that this sample size has a power of 0.80 to detect a medium effect size, *d* = 0.55.

#### Procedure

Participants were randomly assigned to the experimental (*n* = 53) or the control condition (*n* = 55). Prior to administrating the SI questions, only those in the experimental condition were shown the performance criterion, teamwork, and the behavioral items that operationally define it on a BOS. All participants in the two conditions answered the nine predictively valid SI questions on their respective computers. The questions, taken from Klehe and Latham (2005), included a dilemma. A sample question is: “Your group is working on a very important project. All of you want to achieve a good grade. You have a tight deadline. One member of your group was especially successful in this area last term. Supported by two other group members, she takes the lead on your group project. She keeps the minutes and controls the flow of information during the discussion. However, you have the strong impression that she only records ideas supporting her position, and makes decisions on issues without consulting others. What would you do in this situation?” There were no time limits for responding to the questions. The participants were then debriefed and compensated.

All responses to the nine SI questions (*M* = 2.39, *SD* = 0.63) were scored independently by a Ph.D. psychologist and a Ph.D. student in human resource management. Both individuals were blind to the hypothesis and the experimental conditions. The scoring was done using the behavioral scoring guide developed by Klehe and Latham (2005). These two raters were familiar with the SI's behavioral scoring guide. Nevertheless, they practiced the rating process as a dyad before scoring the actual responses to the SI questions independently.

### Results

The *ICC* (3) of the SI was 0.81 indicating adequate inter-rater reliability. The final score for each participant was the average of the scores from the two independent raters. Following the scoring guide by Klehe and Latham (2005), the rating for each SI question ranged from 1 (*unacceptable*) to 5 (*outstanding*). An independent sample two-tailed *t*-test revealed that scores on the SI did not differ significantly between the experimental (*M* = 2.45, *SD* = 0.62) and the control (*M* = 2.34, *SD* = 0.64) group [*t*_(106)_ = 0.89, *p* = 0.37, *d* = 0.17]. We then tested for any effect that an individual's sex, age, years of work experience, number of hours worked per week, and education may have had on this result. No significant effect was found.

### Discussion

The results of this experiment show that having been informed of the performance criterion that the SI was assessing did not enable participants to obtain higher scores on the SI than participants who were not given this information. An arguable limitation of this experiment is that because the participants read the SI questions and wrote their answers to them, the context was not similar to an interview. An additional limitation was that the sample size had low statistical power to detect small effect sizes (*d* < 0.50). Yet, the magnitude of the effect size obtained in this experiment, *d* = 0.17, is relatively negligible and thus provides support for the hypothesis that knowledge of the criterion being assessed has little or no effect on the score in a valid situational interview, namely, SI questions that contain dilemmas.

## Experiment 2

A second experiment was conducted where each participant was interviewed by two interviewers who recorded and scored the answers independently. The purpose of this second experiment was to determine whether the results of the first experiment would be replicated when the participants were actually interviewed.

### Method

#### Participants

The participants were 100 undergraduate students at a large University in Canada (*M*_age_ = 20.75, *SD* = 3.79, 59.8% female). Of this number, 64% were unemployed, 35% worked in a part-time job, and 1% worked in a full-time job. Because the initial data collection was limited to the M.B.A students enrolled in classes taught by the first author, we followed feasibility analysis recommendations to recruit as many participants as possible (Lakens, 2021). A sensitivity power analysis using G*Power (Faul et al., 2007) indicated that this sample size has a power of 0.80 to detect a moderate effect size (*d* = 0.57) in a between-participant design with two groups.

#### Procedure

The interviewees were randomly assigned to the experimental (*n* = 50) or the control (*n* = 50) condition. As in the first experiment, the participants in the experimental condition were shown the performance criterion and the behavioral items that define it, whereas those in the control condition did not receive this information. As in Experiment 1, the interviewees answered the nine valid SI questions from Klehe and Latham (2005), and the interviewers used the same behavioral scoring guide. All participants in the two conditions answered the nine predictively valid SI questions on their respective computers. The responses to the SI questions (*M* = 2.42, *SD* = 0.62, range 1–5) were scored independently by the same two interviewers as in Experiment 1, namely, a Ph.D. psychologist and a Ph.D. student in human resource management who were blind to the hypothesis and the experimental conditions.

### Results

The inter-rater reliability of the answers to the SI was high *ICC* (3) = 0.94. As in the first experiment, there was no significant difference in the SI scores between the experimental (*M* = 2.56, *SD* = 0.48) and the control group (*M* = 2.44, *SD* = 0.46); [*t*_(98)_ = 1.33, *p* = 0.19, *d* = 0.27]. We conducted an additional analysis to examine whether the lack of a main effect on the SI changed when controlling for demographics variables. Specifically, an ANCOVA controlling for an individual's sex, years of employment, and age did not change the conclusion [*F*_(1, 92)_ = 1.89, *p* = 0.17].

### Discussion

This second experiment replicated the results obtained in the previous experiment and hence provides additional support for the conclusion that providing knowledge of the criterion predicted by the SI does not improve the final score when the SI questions include a dilemma. However, these two experiments did not directly compare the two types of questions, namely, those with and without a dilemma. Consequently, we conducted a third experiment since recent SI studies have omitted the dilemma in the interview questions (Taylor and Small, 2002; Huffcutt et al., 2004).

An additional limitation of experiments 1 and 2 is that they did not have sufficient power to detect the average effect size, *d* = 0.22. Nevertheless, this effect size is quite small. It supports the hypothesis that knowledge of the criterion being assessed has little or no effect on an individual's score in a valid situational interview, that is, SI questions that include a dilemma.

## Experiment 3

Because experiments 1 and 2 yielded essentially the same results regardless of whether the SI was administered verbally or in written form, we allowed participants in this third experiment to read the nine SI questions which were presented in the first two experiments and write their answers to them. There was no time limit for doing so.

The second hypothesis of our research that was tested in this third experiment was that the scores of responses to interview questions that do not include a dilemma are significantly higher than scores of responses to the same interview questions that do contain a dilemma.

### Method

#### Participants

We recruited 284 participants through Prolific Academic platform. Prolific academic is an online crowdsourcing platform designed for academic research. This platform includes ~12,000 international participants who participate in scientific studies in exchange for cash rewards for themselves or for one of two chosen charities (Save the Children and Cancer Research UK). Participants in this platform can be selected for a study on the basis of their age, fluent language skills, and approval rate in previous studies.

Four participants wrote meaningless words and combinations of letters as answers to the interview questions and hence were excluded from the data analysis. Thus, the final sample was 280 (*M*_age_ = 34.70, *SD* = 9.13, 51.4% female). Of this number, 94.6% of the participants were employed. Sensitivity analysis indicated that the smallest effect size that this sample size can detect for an interaction with a power of 0.80 in a between-participant 2 X 2 factorial design is *Cohen's f* = 0.20 (Faul et al., 2007).

#### Procedure

Participants were randomly assigned to one of four conditions. Specifically, we crossed the dilemma conditions (yes/no) with knowledge of the criterion conditions (yes/no). Participants in the first condition (*n* = 68) were given the same nine SI questions used in the previous two experiments (*M* = 3.04, *SD* = 0.50). They were informed of the performance criterion that was being assessed by those questions (i.e., dilemma/knowledge of the criterion provided). Participants in the second condition (*n* = 69) were given the nine SI questions that contained a dilemma. They were not informed of the criterion that the questions predicted (i.e., dilemma/no knowledge of the criterion provided). Participants in the third condition (*n* = 72) were given the nine SI questions with no dilemma. They were informed of the performance criterion being assessed (i.e., no dilemma/knowledge of the criterion provided). Participants in the fourth condition (*n* = 71) were given the SI questions that did not include a dilemma. They were not informed of the criterion that the questions predicted (i.e., no dilemma/no knowledge of the criterion). The coders rated the answer to each SI question on a scale ranging from 0 (*unacceptable*) to 5 (*outstanding*). We used the average score for all SI questions across the two ratings.

After the participants responded to the nine SI questions, they answered demographic questions and were compensated $1.60. Two judges independently scored the responses. These judges were M.B.A students who received training on the coding procedure. The inter-rater reliability of the answers to the SI was high, as indicated by *ICC* (3) = 0.84. Therefore, the score for each SI question was the average rating by the two independent judges who were blind to the research hypotheses and experimental conditions. The training of the two judges included an explanation about the SI and how to use the behavioral scoring guide. Both judges were experts in human resource management and were highly knowledgeable of SI procedures prior to participating in this experiment.

### Results

Table 1 presents the descriptive statistics by experimental condition. There was a significant main effect of the dilemma manipulation on SI scores [*F*_(1, 276)_ = 85.30, *p* < 0.001, η^2^_*p*_ = 0.24]. Participants in the no-dilemma condition (*M* = 3.28, *SE* = 0.04) scored significantly higher on the SI than participants in the dilemma condition (*M* = 2.80, *SE* = 0.04). There was no main effect for the knowledge of the criterion manipulation [*F*_(1, 276)_ = 1.38, *p* = 0.241, η^2^_*p*_ = 01]. Those participants who received knowledge of the performance criterion the SI questions were assessing, teamplaying (*M* = 3.07, *SE* = 0.04), did not score significantly higher than the participants who did not have this knowledge (*M* = 3.01, *SE* = 0.04).

**Table 1:** Experiment 3: descriptive statistics by condition.

	**No knowledge**		**Knowledge**	
**Dilemma**	***Mean***	***SD***	***Mean***	***SD***
No	3.20	0.43	3.36	0.42
Yes	2.82	0.43	2.78	0.45

**Figure 1:**
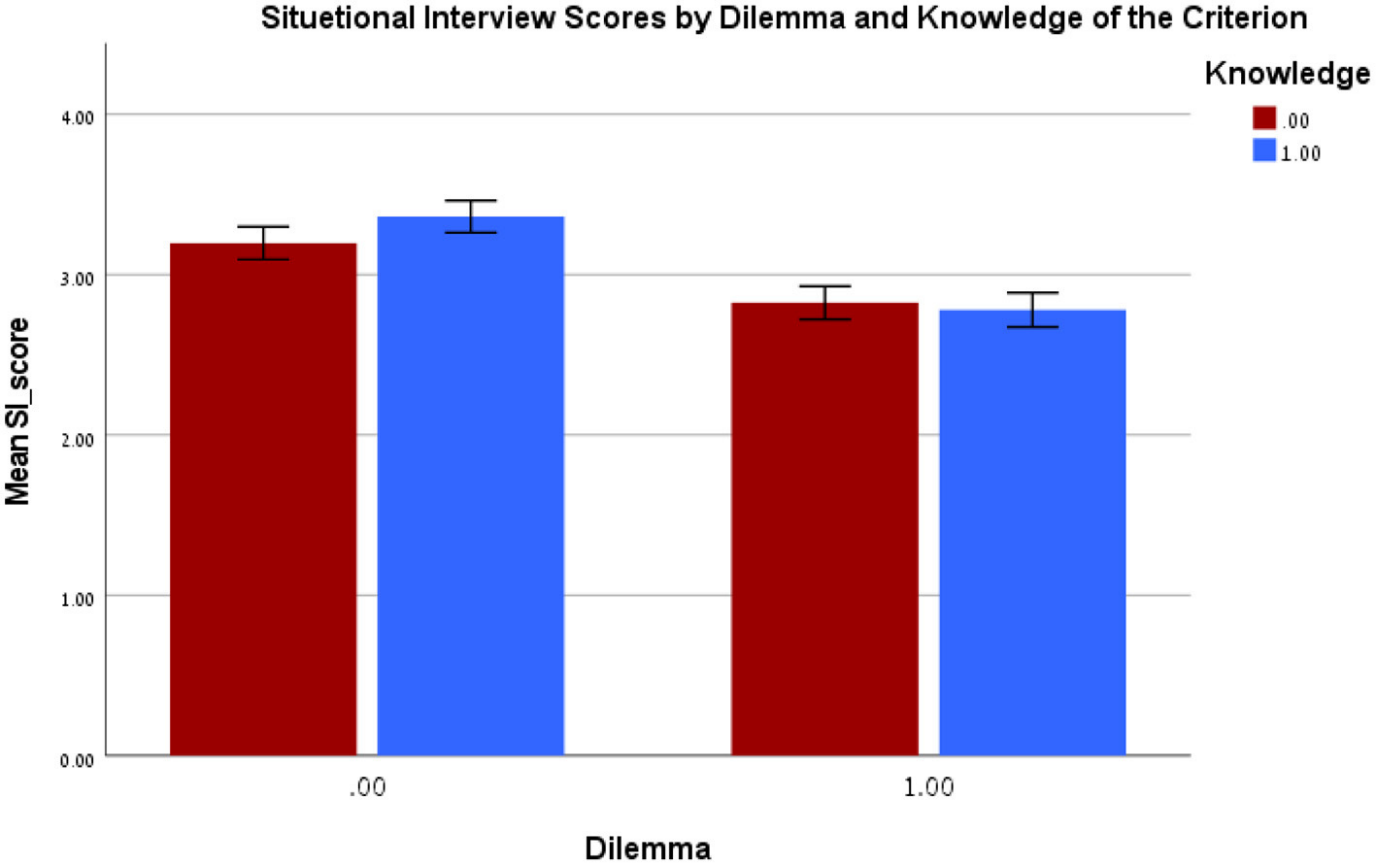
Experiment 3: Interaction between the experimental conditions on Situational interview score. Knowledge 0-no knowledge of the criterion, Knowledge 1-knowledge of the criterion, Dilemma 0-no dilemma, Dilemma 1-dilemma.

There was a significant Dilemma X Knowledge of criterion interaction [*F*_(1, 276)_ = 4.13, *p* = 0.043, η^2^_*p*_ = 0.02] (see Figure 1). Simple effect analysis using the *LSD* test indicated that the interaction was the result of the dilemma manipulation. When the SI questions did *not* include a dilemma, the participants who were provided knowledge of the criterion the interview questions had been developed to assess scored higher on the SI than those who did not receive this information (*M*_difference_ = 0.17, *SE* = 0.07, *p* = 0.024, 95% *CI* [0.02, 0.31]). When the questions did contain a dilemma, there was no significant difference in SI scores between those in the knowledge/no knowledge of criterion conditions (*M*_difference_ = 0.04, *SE* = 0.07, *p* = 0.546, 95% *CI* [−0.19, 0.10]).

### Discussion

The results of this third experiment provide support for the research by Kleinmann and colleagues (e.g., Ingold et al., 2015). When SI questions lack a dilemma, as was the case with the majority of questions used in their experiments, knowledge of the criterion that is being assessed improved scores on the SI. However, the results of the present experiment also provide support for the two preceding experiments. When the SI questions contained a dilemma, knowledge of the performance criterion did not affect SI scores.

## Experiment 4

Subsequent to the Ingold et al. (2015) experiment, Oostrom et al. (2016) arguably used the most rigorous design to date to examine the ATIC-SI relationship. Consistent with the underlying premise of the SI, they found that there was considerable similarity between what participants said they would do, that is, their intentions, and their subsequent behavior. Consistent with previous research on the ATIC, Oostrom et al. (2016) also found that differences in the ability to identify the performance criterion that was being assessed explain why the SI has criterion-related validity.

Once again, the majority of the SI questions used in the Oostrom et al. experiment failed to include a dilemma. Consequently, we modified their questions to include dilemmas. Hence, the purpose of this fourth experiment was 3-fold. First, we again examined scores on the SI where questions did/did not include a dilemma to determine the effect on SI scores. Second, in the three preceding experiments, the SI questions were developed to predict a single criterion, teamplaying. Research on the ATIC sometimes use multiple performance criteria. Hence, we used the same SI questions that assessed the same multiple criteria used by Oostrom et al. (2016).

Finally, none of our three preceding experiments included an explicit measure of ATIC. Note, however, that in the fourth cell of the third experiment the SI questions did not contain a dilemma and the participants were not informed of the criterion that was being assessed. Even though they were not asked to identify the performance criterion, they were free to do so. Yet the participants in this condition did not perform better than those in the other three conditions. Consequently, ATIC was assessed in this fourth experiment. This was done because the ability to identify the performance criteria that are being assessed, after being explicitly asked to do so, may be far different from being informed prior to an SI interview of the performance criteria that are being assessed. Hence, the third hypothesis tested in this fourth experiment is that the ability to identify the performance criteria, when asked to do so, results in SI scores that are significantly lower when the SI questions include a dilemma compared to scores for SI questions that do not include a dilemma.

### Method

#### Participants

We recruited 151 undergraduate students from a college in Israel to participate in this study in exchange for course credit.

Three participants wrote meaningless answers to the SI questions and thus were excluded from the data analysis. The final sample was 148 (*M*_age_ = 22.27, *SD* = 4.39, 48.6% female). Of this number, 23.0% of the participants worked in the private sector, 19.6% in the public sector, and 6.8% were self-employed. The participants had been working, on average, for 3.76 years (*SD* = 4.55). Statistical power was calculated by converting the interaction effect in the third experiment to a Cohen's *f*, which yielded a score of 0.14. Power analysis using GPower indicated that a sample size of 148 participants has a power above 80% to detect an interaction in a regression. Moreover, sensitivity analysis indicated that the smallest interaction effect that this sample size can detect with a power of.80 is $R_{change}^{2}$ = 0.047.

#### Procedure

Participants were informed that they would be taking part in a study about the job interview. To motivate participants to perform well, they were informed that those who received the highest rating would receive an award of 350 NIS (equivalent to $110 US). The experiment, conducted using Qualtrics software, consisted of two parts. In the first part, we manipulated the dilemma.

Participants were randomly assigned to a no-dilemma condition (*n* = 72). They received the 10 SI questions taken from Oostrom et al. (2016). The questions assessed self-control, customer focus, persuasiveness, person-oriented leadership, and task-oriented leadership. The other half of the participants (*n* = 76) received the same SI questions. Each question contained a dilemma that was inserted by the present authors. The interview questions were presented orally to the participants.

An example of an SI question in the no dilemma condition was:

“You have submitted an offer to a customer. You know that you are not the only company that is making an offer. The client has demanded more and more work from you when drawing up the offer. Hence, you believe you will receive the assignment.

You are now with the client who says: “Unfortunately, you did not get the job.” What would you do in this situation?

An SI question with a dilemma was:

“You have submitted an offer to a customer. You know that you are not the only company that is making an offer. The client has demanded more and more work from you when drawing up the offer. Hence, you believe you will receive the assignment. Because this is a big client who demands much of your time, and you were sure you would get the job, you turned down other job opportunities to find the necessary time for this client.

You are now with the client who says: “Unfortunately, you probably won't get the job.” However, if you cut the price by 30%, you might get it.” What would you do in this situation?

In the second part of this experiment, the ATIC was assessed. Consistent with Oostrom et al. (2016), the participants were presented with the following information:

“During the interview, you probably thought about which skills or characteristics were measured by the different questions. We would like to know, for each question, the skills/characteristics that were being measured. Also, please provide concrete behavioral examples that are related to the skills/properties you identified.”

The participants were then presented with each SI question that had been asked of them. They were requested to write the performance criterion that they believed each question assessed, along with a behavioral example. Finally, the participants answered demographic questions before being debriefed.

Two judges who were blind to the research hypotheses and experimental conditions rated the answers to each SI question on a 1-5 Likert-type scale. Specifically, following Oostrom et al. (2016), the categories were labeled as follows: 1- *Not effective at all*, 2- *Not effective, 3- A bit effective*, 4- *Effective, 5- Very effective*. The ATIC answers were evaluated on a 0–3 scale with the following labels: 0- *No match*, 1- *matches a bit*, 2- *matches*, 3-*completely matches*. Both coders received 6 h of training on the SI and ATIC. One judge was an M.B.A student specializing in human resource management. The other judge had an M.B.A. degree and worked as an organizational consultant in the field of employee selection. Both judges were unaware of the hypotheses or the experimental conditions.

The training procedure for the two judges is consistent with prior work on the ATIC and SI (e.g., Oostrom et al., 2016). The training began with an introduction to the SI, the ATIC, and the scoring procedures for each one. The judges were given a behavioral scoring guide for determining whether an answer to each SI question was poor, adequate, or highly acceptable. A practice session enabled the judges to become familiar with the rating process for the SI questions and the ATIC. Afterwards, they discussed their ratings with each other, received feedback on their ratings, and were invited to ask questions of the researchers about the rating process before the experiment began.

### Results

A one-way random effect *ICC* was calculated in order to determine inter-rater reliability. The *ICC* (3) for the SI questions presented was 0.88, and the correlation between the two judges was 0.79. The *ICC* (3) for the ATIC questions was 0.92, and the correlation between the judges was 0.88. Therefore, both the SI and the ATIC scores were calculated as the mean rating of the two judges.

Table 2 presents the descriptive statistics and correlations among the study's variables.

**Table 2:** Experiment 4: descriptive statistics and correlations.

	***M***	***SD***	**1**	**2**	**3**	**4**
1. ATIC	1.07	0.29				
2. SI	2.46	0.58	0.33^**^			
3. Work experience (in years)	3.76	4.55	0.01	−0.03		
4. Age	22.27	4.39	0.01	0.07	0.84^**^	
5. Gender	1.49	0.50	−0.02	0.15	−0.07	−0.01

** *p < 0.01; Gender was coded as 1- female, 2- male*.

#### Main Effects

There was a significant main effect of the dilemma manipulation on SI scores [*t*_(146)_ = 8.29, *p* < 0.001, *d* = 1.36]. Participants in the no-dilemma condition (*M* = 2.80, *SD* = 0.54) scored significantly higher on the SI than participants in the dilemma condition (*M* = 2.14, *SD*= 0.42). There was no main effect of ATIC between the experimental groups [*t*_(146)_ = 1.16, *p* = 0.25, *d* = 0.19]. Participants in the dilemma condition (*M* = 1.04, *SD* = 0.24) did not differ on their ATIC score relative to participants in the no-dilemma condition (*M* = 1.09, *SD* = 0.34).

#### Moderation Analysis

**Figure 2:**
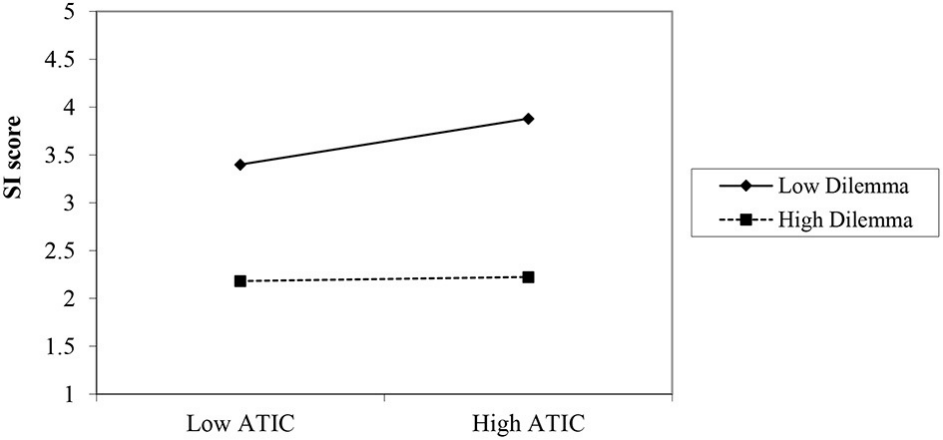
Experiment 4: Interaction of the relationship between SI and ATIC by experimental condition.

We conducted a moderation analysis using Model 1 in PROCESS (Hayes, 2017) using 5,000 bootstrapped samples. First, we centered the ATIC scores on its means. The results indicated that the ATIC had a main effect on the SI score—controlling for the other main effect and the interaction [*b* = 0.82, *SE* = 0.16, *t* = 5.22, *p* < 0.001]. The manipulation had a main effect controlling for ATIC score [*b* = −0.63, *SE* = 0.07, *t* = −8.59, *p* < 0.001]. In addition, as hypothesized, ATIC was associated with higher scores on the SI interview only in the no dilemma condition. Specifically, the Manipulation X ATIC interaction had a significant effect on SI, controlling for the main effect of the manipulation and the ATIC was significant [*b* = −0.73, SE = 0.26, *t* = −2.78, *p* = 0.006], $R_{change}^{2}$ = 0.03, *F*_(1, 144)_ = 7.76, *p* = 0.006]. As shown in Figure 2, a simple slope analysis indicated that in the no dilemma condition, ATIC significantly increased performance in the SI [*b* = 0.82, *SE* = 0.16, *t* = 5.22, *p* < 0.001]. However, the ATIC did not increase SI scores when a dilemma was included in the interview questions [*b* = 0.08, *SE* = 0.21, *t* = 0.38, *p* = 0.70]. Put differently, the association between ATIC and scores on the SI was strong, positive and significant only in the no-dilemma condition [*r*_(72)_ = 0.51. *p* < 0.001], yet it was not significant in the dilemma condition, [*r*_(75)_ = 0.05, *p* = 0.69].

### Discussion

The results of this fourth experiment provide additional support for the first three experiments as well as support for previous research on the ATIC. Specifically, when SI questions lacked a dilemma, the ATIC increased scores on the SI in this fourth experiment. This finding replicates previous work on this topic (e.g., Ingold et al., 2015; Oostrom et al., 2016). However, when a dilemma was included in each SI question, the ATIC score did not increase scores on the SI. The findings of this fourth experiment enhance the external validity of the findings in the three previous experiments by assessing multiple performance criteria as well as using the same interview questions that were used in Oostrom et al.'s (2016) research, and in addition including a condition where individuals were requested to identify the performance criteria that the questions predicted.

## General Discussion

The present four experiments are similar to Ingold et al.'s (2015) in that the participants in the first two experiments and the participants in the Oostrom et al. (2016) study were, or had been, employed in a variety of sectors. The SI used in our four experiments and the SI used by Ingold et al. and Oostrom et al. (2016) had criterion-related validity. The inter-rater reliability estimates in the present four experiments are similar to the reliability estimates obtained by both Ingold et al. (2015) and Oostrom et al. (2016).

The differences between the Ingold et al. and Oostrom et al. studies vs. the present experiments are at least 2-fold. First, the SI questions in the present research, as shown in the Appendices, contained dilemmas whereas the majority of questions used by Ingold et al. and Oostrom et al. (2016) failed to do so. In addition, we informed participants in the experimental group in experiments 1–3 of the performance criterion that was being assessed, whereas Ingold et al. and Oostrom et al. required participants to guess the performance criterion on which they were being assessed. However, this was also the case in our fourth experiment. Individuals in the experimental condition were asked to identify the criteria that was being assessed.

The results of our first experiment were replicated in the second and third experiments. These results suggest that knowing the performance criterion that is being assessed is not advantageous for attaining higher scores on a SI if the SI questions contain a dilemma. Furthermore, the results of the fourth experiment, which included a measure of ATIC, showed that it is the existence of a dilemma in SI questions that disentangles the relationship between the ATIC and SI. When the SI questions in our fourth experiment did not include a dilemma, the ATIC significantly increased SI scores, thus replicating the findings of both Ingold et al. (2015) and Oostrom et al. (2016).

The practical significance of the present four experiments, in addition to casting doubt on the relevance of ATIC to a correctly developed SI, is that it shows the necessity of adhering to a critical step required for developing a SI, namely including a dilemma in each question. Had this been done by Ingold et al. (2015), they would likely have obtained findings similar to that of our first and second experiments. The present findings hopefully shed light on an important element in the development of an SI, namely, “the dilemma.” The inclusion of a dilemma appears to have been lost from this technique in its purer form in myriad studies. Researchers and practitioners should refrain from what is becoming a common practice (see Taylor and Small, 2002; Huffcutt et al., 2004), namely, treating SI questions with and without a dilemma as interchangeable. If this recommendation is followed, managers can remain confident that the SI is a reliable and valid technique for selecting employees.

## Limitations and Future Research

The limitations of our research are at least 3-fold. Arguably the criterion, teamwork, used in three of our four experiments may have been readily discerned by the content of the SI questions. If this argument has merit, the participants in the control condition might have been able to easily guess the criterion that was being assessed. However, if this explanation were correct, Klehe and Latham (2005) would not have obtained evidence of predictive validity for the SI, due to restriction of range as the majority of the participants would have been able to give socially desirable answers. Moreover, we would not have found a significant interaction effect in our third experiment. Note too that consistent with Oostrom et al. (2016), multiple performance criteria were used in our fourth experiment.

An arguable second limitation of the first three experiments, as noted earlier, is that making people aware of the performance criterion is not the same as what is presumed to be an individual difference variable, namely, ATIC. Nevertheless, the results from the fourth experiment replicated the findings by Ingold et al. in the no-dilemma condition. The results also replicated the findings of our third experiment. The ATIC did not enhance SI scores in the dilemma condition.

To further investigate whether ATIC is different from providing individuals with knowledge of the criteria being assessed, future research should use a 2 × 2 factorial design crossing knowledge of the criterion and a dilemma along with a measure of ATIC. Such an experiment will provide information about the incremental validity of the ATIC relative to knowledge of the criterion that is being assessed by valid SI questions that is, those that contain dilemmas.

Note that the gold standard for adequately questioning the results obtained by other researchers, in this instance, Ingold et al. (2015) and Oostrom et al. (2016), involves a two-step process. First, skeptics must show that they can replicate the original findings. Second, they must show that those findings are due to a methodological artifact, in this instance, the absence of a dilemma in an SI question. Only questions that contain a dilemma warrant the designation of SI. These two steps were taken in the present research.

A third limitation concerns the issue of statistical power. The third and fourth experiments had adequate power, namely above 80% to detect a medium effect size. However, the effects in previous studies on ATIC and the SI found small effect sizes (e.g., Klehe et al., 2008). Hence, future studies should use large sample sizes to further explore the effect of a dilemma in the ATIC-SI relationship.

A fourth arguable limitation is that the difficulty level of the questions in the dilemma vs. no-dilemma conditions was not held constant. If this criticism were appropriate, previous research comparing the criterion-related validity of structured vs. unstructured interviews, the latter often involving little more than a casual conversation, would be redacted. Structured interviews that are job-related are typically more difficult for a job applicant than participating in a free-flowing unstructured discussion. Similarly, including a dilemma in an SI question makes it more difficult to answer than it is to respond to questions that lack a dilemma. A dilemma is an inherent quality of the SI (Levashina et al., 2014).

In summary, the issue underlying this paper is that many practitioners and researchers have ignored the recommendations of Latham (1989) for constructing SI questions. The primary finding of the present research is that ability to identify the criteria being assessed by an SI increases an individual's score only when the questions fail to contain a dilemma.

## Data Availability Statement

The raw data supporting the conclusions of this article will be made available by the authors, without undue reservation.

## Ethics Statement

The studies involving human participants were reviewed and approved by University of Toronto. The patients/participants provided their written informed consent to participate in this study.

## Author Contributions

GL and GI contributed to the conception and design of the studies. GI performed the statistical analysis and wrote the method and results were written jointly by the authors. GL wrote the introduction and discussion. All authors approved the submitted version.

## Conflict of Interest

The authors declare that the research was conducted in the absence of any commercial or financial relationships that could be construed as a potential conflict of interest.

## Publisher's Note

All claims expressed in this article are solely those of the authors and do not necessarily represent those of their affiliated organizations, or those of the publisher, the editors and the reviewers. Any product that may be evaluated in this article, or claim that may be made by its manufacturer, is not guaranteed or endorsed by the publisher.

## Footnotes

^1^Another arguable advantage of including a dilemma in SI questions is that doing so increases their difficulty level for interviewees.

^2^In addition to using interview questions that did not include a dilemma, Pulakos and Schmitt (1995) also made the performance criterion transparent to the interviewees.
