# Supplementary material for: The Effect of a Dilemma on the Relationship Between Ability to Identify the Criterion (ATIC) and Scores on a Validated Situational Interview
Source: Front Psychol. 2021 Jul 27;12:674815. doi: 10.3389/fpsyg.2021.674815 (PMC8353324; doi:10.3389/fpsyg.2021.674815)
Supplement: Supplementary file 1 [file Data_Sheet_1.docx]

**Appendix A**

**Experiment 3: Examples of scenarios**

**No-dilemma condition**

Your study group has to complete an important project. It is important for each of you to do very well on this project. You have one group member who is willing to work hard and whose capabilities in this area are very high. Your group is under high time pressure. What would you do in this situation?

**Dilemma condition**

Your study group has to complete an important project. It is important for each of you to do very well on this project. You have one group member who is willing to work hard but whose capabilities in this area are very low. Your group is under high time pressure. What would you do in this situation?

**Appendix B:** (translated from Hebrew): Examples of SI questions in Experiment 4

**No dilemma condition**

1. You have submitted an offer to a customer. You know that you are not the only company that makes an offer. The client has demanded more and more work from you when drawing up the offer, and you have thereby been given the impression that you will receive the assignment. You are now with the client. The client says:” “Unfortunately, you did not get the job.” What would you do in this situation?
2. You are a salesperson, and you have the first meeting with a potential customer. You have talked about the needs of the customer and about the possibilities of your company to realize those needs. You come to the end of your conversation. The customer responds:” “Good, clear! Thank you for your efforts to come and tell us about what you have to offer.” (is getting ready to stand up and say goodbye) What would you do in this situation?

**Dilemma condition**

1. Your boss has stressed the importance of losing no more competitive bids without decreasing price. You have submitted an offer to a customer. You know that you are not the only company that makes an offer. The client has demanded more and more work from you when drawing up the offer, and you have thereby been given the impression that you will receive the assignment. Because this is a big client who demands much of your time and you were sure you will get the job, you turned down other job opportunities to clear time for this client.  
    You are now with the client. The client says:” “Unfortunately, you probably ’won’t get the job.” Unless you cut the price by 30%, you will get it.   What would you do or say?
2. You are a salesperson, and you have the first meeting with a potential customer. You have talked about the needs of the customer and about the possibilities of your company to realize those needs. You come to the end of your conversation.
    Customer:” “Good, clear! Thank you for your efforts to come and tell us about what you have to offer, I’m late for a very important meeting”. (Is getting ready to stand up and say goodbye). Suddenly you realize you forgot to mention a very important detail that can make your offer extremely compelling but takes time to explain.  What would you do in this situation?
